# Thermostable Vaccines in Veterinary Medicine: State of the Art and Opportunities to Be Seized

**DOI:** 10.3390/vaccines10020245

**Published:** 2022-02-05

**Authors:** Angela Fanelli, Luca Mantegazza, Saskia Hendrickx, Ilaria Capua

**Affiliations:** 1 One Health Center of Excellence, University of Florida, Gainesville, FL 32603, USA; angela.fanelli@uniba.it (A.F.); mantegazza@ufl.edu (L.M.); 2 Department of Veterinary Medicine, University of Bari, 70010 Valenzano, Italy; 3 Feed the Future Innovation Lab for Livestock Systems, University of Florida, Gainesville, FL 32603, USA; scjhendrickx@ufl.edu

**Keywords:** thermostable, heat-stable, freeze-stable, vaccines, COVID-19 legacy, vaccine efficacy

## Abstract

The COVID-19 pandemic has highlighted the weakness of the vaccine supply chain, and the lack of thermostable formulations is one of its major limitations. This study presents evidence from peer-reviewed literature on the development of thermostable vaccines for veterinary use. A systematic review and meta-analysis were performed to evaluate the immunogenicity and/or the efficacy/effectiveness of thermostable vaccines against infectious diseases. The selected studies (*n* = 78) assessed the vaccine’s heat stability under different temperature conditions and over different periods. Only one study assessed the exposure of the vaccine to freezing temperatures. Two field studies provided robust evidence on the immunogenicity of commercial vaccines stored at temperatures far in excess of the manufacturer’s recommended cold-chain conditions. The drying process was the most-used method to improve the vaccine’s thermostability, along with the use of different stabilizers. The pooled vaccine efficacy was estimated to be high (VE = 69%), highlighting the importance of vaccination in reducing the economic losses due to the disease impact. These findings provide evidence on the needs and benefits of developing a portfolio of heat- and freeze-stable veterinary vaccines to unleash the true potential of immunization as an essential component of improved animal health and welfare, reduce the burden of certain zoonotic events and thus contribute to economic resilience worldwide.

## 1. Introduction

Global vaccine availability and equity is a goal advocated by global leaders and by 170 Nobel Laureates [1]. Nevertheless, the current COVID-19 pandemic has highlighted that the global vaccine coverage is highly inequitable and skewed, with a high vaccine uptake concentrated in selected countries, predominantly the G7 and European ones [2]. Recently, the G20 Summit has underlined the urgent need to intensify efforts to enhance timely, global, and equitable access to safe, effective, and affordable COVID-19 vaccines [3]. In fact, logistical and supply chain system failures have slowed the vaccine availability and have hampered the global efforts to up-scale COVID-19 vaccination coverage. The lack of thermostability has been proven to be one of the major barriers limiting the worldwide distribution of these products [4]. Indeed, the race to develop efficacious SARS-CoV-2 vaccines has resulted in the first available commercial vaccine products to have storage and delivery requirements of temperatures between +2 °C and −70 °C, depending on the product [5,6,7,8].

It is surprising that in 2021 the vast majority of vaccines for human and animal diseases are still dependent on cold-chain systems to ensure their potency throughout production, shipment, storage, and administration. In both human and animal health, vaccines resistant to damage by heat and freezing could have great economic and health benefits. Heat-freeze-stable vaccines could help to reduce vaccine wastage and prevent the consequences of administering ineffective vaccines [9]. For these reasons, thermostable vaccines have been named a priority research area in the World Health Organisation’s Global Vaccine Action Plan 2011–2020 [10]. Nevertheless, their development and production is not always a prime concern for vaccine developers, industries, and funding entities [2].

Vaccination is an effective preventive measure against infectious diseases. The main objective of livestock vaccines is to improve animal health, reducing the economic losses associated with disease occurrences [11]. The use of vaccines is recognised as an important management option during outbreaks, as it helps to control the spread of infection and reduce the need for the large-scale culling of at-risk animals [12]. Vaccines are also essential to sustain the commercial exchange of animal products between countries. Vaccines have been developed for 53% (63/117) of the OIE listed diseases (Appendix A: Table A1) [13,14], while the production of vaccines has been historically reported by members to the World Organisation for Animal Health (OIE) for 68 diseases. When considering these data, it should be noted that only the laboratories under national veterinary services are requested to provide information to the OIE on the vaccines produced (e.g., vaccines produced by private firms are not reported to the OIE) (Appendix A: Table A2).

Most vaccines require continuous storage at 2–8 °C from manufacturing through to administration, requiring a cold-chain system for their transportation [15]. Vaccination campaigns for several OIE-listed diseases (e.g., Foot-and-mouth disease (FMD) and Rabies) are highly encouraged in endemically infected countries to combat disease outbreaks and reduce their economic burden [16]. These are generally low- and middle-income countries which do not have widespread access to a stable supply of electricity or an effective cold-chain system for vaccines. Considering this, thermal stability is a critical issue for most of the available vaccines against animal diseases of international concern. 

Similarly, half of the supplied vaccines for human use are wasted as a result of inadequate cold-chain capacities [15]. It has been estimated that this loss accounts for about 80% of the total cost of vaccination programs, which is roughly $200–$300 million per year [17]. In the worst circumstances, the damages may remain undetected, increasing the chance that vaccines with reduced potency are administered, exposing the recipients to a higher risk of becoming infected or even ill [18]. There are no such studies for veterinary vaccines, but we can assume similar figures. For these reasons, it would seem reasonable to invest in solutions that can address the core fragilities which are embedded in most vaccines that are on the market today. Indeed, we have previous experiences which underscore the importance of having heat-stable vaccines.

To date, Rinderpest in cattle, and smallpox in humans, are the only diseases that have been officially eradicated. For both diseases, indispensable to the success of the eradication was the adequate supply of heat-stable and potent vaccines [19,20]. The benefits of developing thermostable vaccines for humans were reviewed by several studies [9,21]. Additionally, the economic impact of their use was estimated in different case studies in developing countries. For instance, Lee et al. [22] developed a computational model to simulate the effects of making some vaccines thermostable in Niger. They showed that even a single thermostable vaccine would free significant cold storage space for other vaccines, thus alleviating supply chain bottlenecks. In Benin, another study showed that replacing different existing vaccines with thermostable formulations would save medical costs and productivity losses, even with a price two-to-three times higher than the non-thermostable product [23]. Although no study evaluating the economic impact of thermostable vaccines for veterinary use has been carried out, it is reasonable to assume that it would be significant, especially considering how livestock plays an important role in the economy of developing countries, contributing to the livelihoods of about 1.7 billion people [24]. 

The potential impact of making certain formulations thermostable appears evident when looking at the figures of vaccines commercialized by private companies and authorized by the agencies responsible for the evaluation and supervision of medicines. For instance, the USDA Animal and Plant Health Inspection Service (APHIS) licensed more than 700 vaccines, bacterins, and immunomodulators (USDA APHIS: Veterinary biologics, product summaries: https://www.aphis.usda.gov/aphis/ourfocus/animalhealth/veterinary-biologics/product-summaries, accessed on 25 November 2021), while the European Medicines Agency (EMA) lists more than 400 vaccines approved for animal use (EMA veterinary medicine database: http://vet.eudrapharm.eu/vet/advancedSearch.do, accessed on 25 November 2021). Billions of doses are administered annually to protect the worldwide poultry population (STATISTA: https://www.statista.com/statistics/263962/number-of-chickens-worldwide-since-1990/, accessed on 25 November 2021).

A vaccine that did not require cold temperatures to be transported and stored would eliminate the costs of maintaining the cold-chain and would address equity issues linked to the unavailability of a reliable electricity supply. The positive impact would be also seen in high-income countries, as thermostable vaccines would be easier and cheaper to store. For example, Porphyre et al. [25] identified the importance of sufficient strategic supplies of vaccines to control FMD outbreaks in Scotland. The easy distribution and storage of thermostable vaccines would greatly influence delivery rates and, thus, the reaction timing for controlling outbreaks in livestock. This is particularly true when considering highly contagious diseases, such as FMD [26]. The general consensus is that vaccination is one of the essential tools to respond to outbreaks of livestock diseases which cannot be controlled by stamping-out policies. In these cases, vaccination is also considered the control option that provides the largest economic benefits [27,28].

As an unsurprising starting point, it should be mentioned that the characteristics of thermostable vaccines are not clearly and specifically defined. The World Health Organization (WHO) encourages the production of thermostable vaccines, considering them, in general terms, as heat- and freeze-stable formulations which can be stored for extended periods of time above 8 °C, as well as not being damaged by freezing temperatures (<0 °C) [29]. The OIE, which sets the standards for the production and quality control of biological products for veterinary use across the globe [30], uses the word ‘thermotolerant’ to describe the ability of a vaccine to retain a level of infectivity after exposure to heat (Glossary of terms of the OIE Terrestrial Manual https://www.oie.int/app/uploads/2021/03/mailing-oct-2014.pdf, accessed on 25 November 2021). However, it does not provide a clear definition of thermotolerance or thermostability in terms of its shelf-life and its recommended stability, with reference to temperature ranges. Moreover, the Food and Agriculture Organization of the United Nations (FAO) and the Pan American Health Organization (PAHO), which have high-level scientific and technical expertise from around the world in dealing with priority health issues, do not outline a standard for thermostable vaccines [31,32]. The lack of a standard, as well as a unified definition, from the international organisations involved in the fight against human and animal diseases at a global level, contributes to the hinderance in the production of thermostable formulations.

Today, given the evidence of the inequitable access to vaccines, supply chain challenges, and the continuing rise in new cases of COVID-19, particularly in low- and middle-income countries, the world has a perfect opportunity to identify bottlenecks and to reprioritize research. The transformative power of the COVID-19 pandemic calls for major advancements in vaccine development and manufacturing, which would empower decision makers and the scientific community to unleash the full potential of vaccines and immunization. Considering the above, the objective of this study is to gather, assess, and present evidence from the peer-reviewed literature on thermostable vaccines developed for animal diseases and providing examples of their value, as well as discussing their impact on disease prevention and control. 

## 2. Materials and Methods

### 2.1. Objectives

This systematic review and meta-analysis focus on a selection of animal infectious diseases with the objective of answering the following guiding questions:

1. What type of thermostable vaccines have been developed for veterinary use?
2. What are the characteristics of these thermostable vaccines?
3. How immunogenic and effective are these thermostable vaccines?

### 2.2. Eligibility Criteria

The inclusion criteria are: (1) the clinical and field trials evaluating the immunogenicity and/or the efficacy/effectiveness of thermostable vaccine formulations developed against animal infectious diseases (only bacterial and viral diseases); (2) studies testing thermostable vaccines in natural hosts; (3) articles published in peer-reviewed journals after 1990; and (4) an English language full text. Experimental studies using laboratory animals (e.g., mice) and in vitro studies are excluded.

### 2.3. Information Sources

PubMed, CAB Abstracts, and Web of Science databases were used to perform two separate literature searches: a broad search on thermostable vaccines, and a specific search on DNA vaccines, which are the new-generation vaccines that are considered heat-stable on account of their structural character [11,33,34]. The first search was done using general keywords and was integrated by screening the reference lists of the identified eligible studies. For the search on DNA vaccines, the terms used to label articles (MeSHterms or Subject category) were implemented, and only the titles of the first 100 returns (sorted by relevance) from each database were retrieved, since the timeframe for this study only allowed for a rapid assessment. The decision of performing two separate searches was for the following reasons:

• Authors may not specify that DNA vaccines do not need the cold-chain, a thermostability is an intrinsic characteristic of these vaccines. Thus, the computerized search would not be able to retrieve the manuscripts if it only used general keywords;
• The use of a unique complex search strategy, combining multiple different terms, would not be an efficient way to identify relevant articles.

The last search was done on 8 September 2021.

Details on the search strategies are provided in Table 1. 

### 2.4. Data Collection Process and Data 

Two data extraction sheets were created in Microsoft Excel, version 2017. In the first database, the following information for each study was recorded: the authors, year of publication, target agent, type of agent (bacterium/virus), animal species, country, product name, vaccine type, strain, market availability (locally produced, commercially available, or experimentally developed), thermostability characteristics, route of administration, type of study (clinical or field trial), assessment (objective), test used, main results, and comments. If the data was not provided, ‘N.A.’ (NOT AVAILABLE) was written. If some information was difficult to extract, a comment was written to that cell. The second database was created to retrieve quantitative data from clinical and field trials assessing the vaccine efficacy/effectiveness after its challenge with the infectious organism. The vaccine efficacy was measured in the clinical trial, as well as how well the vaccine performed in controlled settings. On the other hand, the vaccine effectiveness was defined as the measure of how well the vaccine works in the real world and was measured in the field trials. Vaccine efficacy/effectiveness can be computed by estimating the incidence rate of the disease among vaccinated and unvaccinated groups and determining the percentage of reduction in the incidence rate of the disease among vaccinated animals, compared to unvaccinated animals (1-risk ratio) [35,36]. To build this database we only considered the studies on diseases which are severe and sudden in onset (acute conditions leading to death), while studies assessing the morbidity rate were excluded. The following information was retrieved: the number of deaths after challenging in the vaccinated group, the number of survivals after challenging in the vaccinated group, the number of deaths after challenging in the control group, the number of survivals after challenging in the control group, the challenge time (days post-vaccination, dpv), the relative percent of survival (RPS)/days post-challenge (most of the studies computed the relative percentage of survival (RPS) from the cumulative mortalities in the vaccinated group (Mvac) and unvaccinated control (Munvac): RPS = [1 − (Mvac/Munvac)] × 100%). If a single study had data for more than one experimental group, then those studies were considered as separate studies according to the number of the vaccinated groups under investigation. All authors checked the quality of the data extracted. Any disagreement in the results were resolved by discussion within the team.

### 2.5. Risk of Bias (Quality Assessment)

To minimize the risk of bias in individual studies, anything that could have potentially affected the interpretation of the study was written in the comments section of the data extraction sheets.

### 2.6. Method of Analysis

Results were summarised with text descriptions, tables, and waffle graphs. A meta-analysis with a random-effects model was performed, using quantitative data from studies on fatal diseases. The pooled risk ratio (RR) was calculated, along with the corresponding 95% CI, to report the vaccine efficacy (VE). Studies with less than 10 animals per group were excluded. The analysis was done with the ‘meta’ and ‘metafor’ packages in R software version 4.1.1. [37]. The inverse variance index (I^2^) was used to quantify heterogeneity, indicating the I^2^ values of 25%, 50%, and 75% as low, moderate, and high heterogeneity, respectively [38]. Outliers were investigated using the Baujat and diagnostic plots [39,40]. The potential publication bias was assessed by the examination of the funnel plot. Considering that the asymmetry observed in a funnel plot may be also due to the correlation between the log of RR and its SE, the presence of a small study effect was tested with the Peters’ test for binary outcomes [41]. Subgroup analyses, using mixed effect models, were performed to identify possible sources of heterogeneity related to the animal species and the type of agent. 

## 3. Results

### 3.1. Study Selection 

**Figure 1:**
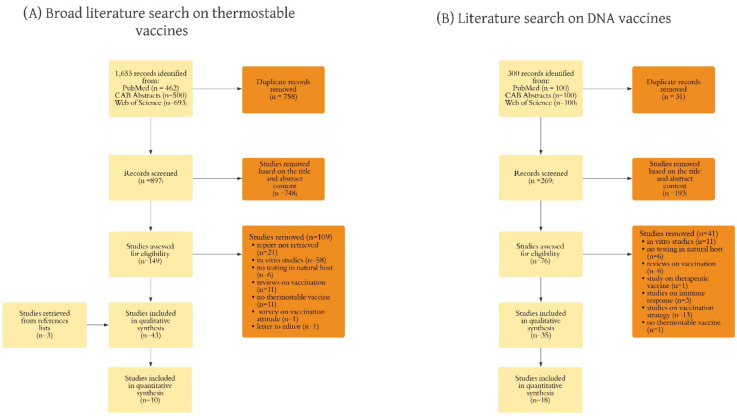
Diagram showing the stepwise process of study selection and pre-determined inclusion and reasons of exclusion for the broad search on thermostable vaccines (**A**), and the literature search on DNA vaccines (**B**).

The first literature search identified a total of 1,655 studies. After the duplicates (*n* = 758) were removed, the titles and abstracts of the remaining studies (*n* = 897) were screened for relevance, and 149 articles were further evaluated for eligibility based on the inclusion criteria. Out of them, 40 were included in the qualitative synthesis, along with three articles retrieved with the screening of the reference lists. Finally, 10 articles were included in the meta-analysis (Figure 1A). Considering the articles on DNA vaccines (*n* = 300), 31 duplicates were removed, and the titles and abstracts of the remaining articles (*n* = 269) were screened for relevance. Seventy-six articles were assessed for eligibility. Out of them, 35 were included in the qualitative synthesis, and 18 were included in the meta-analysis (Figure 1B). 

### 3.2. Study Characteristics

A total of 78 studies, published between 1990 and 2021, were included in this systematic review (Table 2 and Table 3). 

These include:

• Two studies performing both clinical and field trials (one using vaccinated and control groups, and one with all animals vaccinated);
• Thirteen studies performing field trials (eight using vaccinated and control groups, and five with all animals vaccinated);
• Sixty-three studies performing clinical trials (60 using vaccinated and control groups, and three with all animals vaccinated).

**Figure 2:**
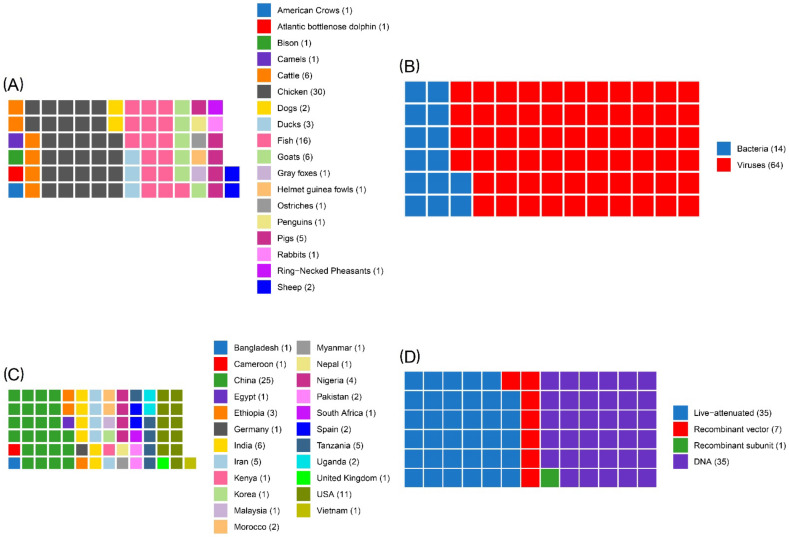
Waffle graphs highlighting the attributes of the studies included: animal species (**A**), type of agent (**B**), country (**C**), and type of vaccine (**D**).

Most of the studies were carried out in chickens (*n* = 30), followed by fish (*n* = 16). Further details on the animal species are provided in the waffle chart (Figure 2A). 

With regards to the type of agent, 64 studies were on diseases caused by viruses and 14 by bacteria (Figure 2B). The majority of the articles were on vaccines against the Newcastle disease virus (NDV) (*n* = 23), followed by the Peste des petits ruminants virus (PPRV) (*n* = 6). The rank order of countries, based on the number of studies retrieved, was China (*n* = 25), USA (*n* = 11), India (*n* = 6), Iran (*n* = 5), Tanzania (*n* = 5), Nigeria (*n* = 4), Ethiopia (*n* = 3) (one study was carried out on cattle in both Ethiopia and Kenya), Morocco (*n* = 2), Pakistan (*n* = 2), Spain (*n* = 2), Uganda (*n* = 2), Bangladesh (*n* = 1), Cameroon (*n* = 1), Egypt (*n* = 1), Germany (*n* = 1), Kenya (*n* = 1)^2^, Korea (*n* = 1), Malaysia (*n* = 1), Myanmar (*n* = 1), Nepal (*n* = 1), South Africa (*n* = 1), United Kingdom (*n* = 1), and Vietnam (*n =* 1) (Figure 2C). In addition to the articles retrieved through searching DNA vaccines (*n* = 35), 35 studies were on live-attenuated vaccines, seven were on recombinant vector vaccines, and one was on a recombinant subunit vaccine (Figure 2D). Most of the vaccines were experimentally developed (*n* = 56), while a few studies used vaccines that were locally produced (*n* = 16) or were available on the market (*n* = 6). 

Out of the 43 articles retrieved with the broad literature search, 13 studies (these include only the studies that explicitly state that the freeze-drying process was used for the vaccine development) implemented a lyophilization (freeze-drying process) to obtain thermostability [47,49,50,52,65,66,67,70,74,76,79,85]. An alternative drying process was applied by Lv et al. [64] and Smith et al. [77], who used the vaporization method (foam-drying) to preserve the live attenuated vaccines against the porcine reproductive and respiratory syndrome virus (PRRSV) and the rabies virus (RABV), respectively, while Dulal et al. [51] successfully used the sugar-membrane technology to thermostabilize an adenovirus-vectored vaccine against the Rift Valley fever virus. 

Thermostability characteristics were not reported in 23 studies (Table 2). 

These include:

• Studies on vaccines against NDV (*n* = 17), specifying the use of thermostable strains [42,43,45,52,53,54,55,56,57,67,69,70,71,75,82,83,84];
• Studies on vaccines against PPRV (*n* = 2), comprising of one work testing a Rinderpest heterologous vaccine [59], and one developing a heat-stable recombinant subunit vaccine expressed in the peanut plant [60];
• Studies (*n* = 2) using *Bacillus thuringiensis* and the surface layer protein CTC as a vaccine delivery system to develop heat-stable vaccines against avian influenza [63] and *Mycoplasma gallisepticum* [62];
• Study on a vaccine against Rinderpest virus (*n* = 1), describing a recombinant heat stable vaccinia virus [80].

The remaining studies assessed the heat stability under different temperature conditions and over different time periods, from 3–4 days at 40 °C [47] to 25 °C for 12 months [64]. Details on each study are provided in Table 2. All the articles on DNA vaccines did not provide information on thermal stability. Nevertheless, some of them mentioned, in the introduction section, that DNA vaccines do not require the maintenance of a cold-chain as they are thermostable (e.g., Bande et al. [88]). Interestingly, only one retrieved study assessed the exposure of the vaccine to freezing temperatures [68]. 

Considering the objective of the study, 27 works aimed to assess humoral immunity, estimating the antibody titres after vaccine administration, and five articles evaluated both humoral and cell-mediated immunities. In the remaining studies (*n* = 46), animals were challenged with an infectious disease organism, evaluating the humoral immunity, cell-mediated immunity, clinical signs, histopathological changes, or survival rates post-challenge. 

Only a few authors reported an insufficient immune response after vaccination. In particular, Rahman et al. [72] described a partial seroconversion in goats after the vaccination against PPRV, and Bunning et al. [89] reported a failure of the oral vaccination with a DNA vaccine against the West Nile virus (WNV) in the American crow.

### 3.3. Risk of Bias (Quality) Assessment

Overall, no relevant comments that could have affected the outcomes of the studies included were identified.

### 3.4. Synthesis of Results 

Twenty-eight studies, comprising of 60 vaccinated groups, were included in the meta-analysis (Table 4). 

The animals in the trials included avian species (*n* = 12) and fish (*n* = 16), while the target agents were the virus (*n* = 18) and the bacteria (*n* = 10). As shown in Table 4, the RPS was lower than 50% in 10/60 vaccinated groups. These include vaccinated groups from studies investigating the suitability and efficacy of different administration routes [43,89,119], strains [71], or doses [105]. It is important to consider that all these studies have at least one vaccinated group with the RPS > 50%. 

**Figure 3:**
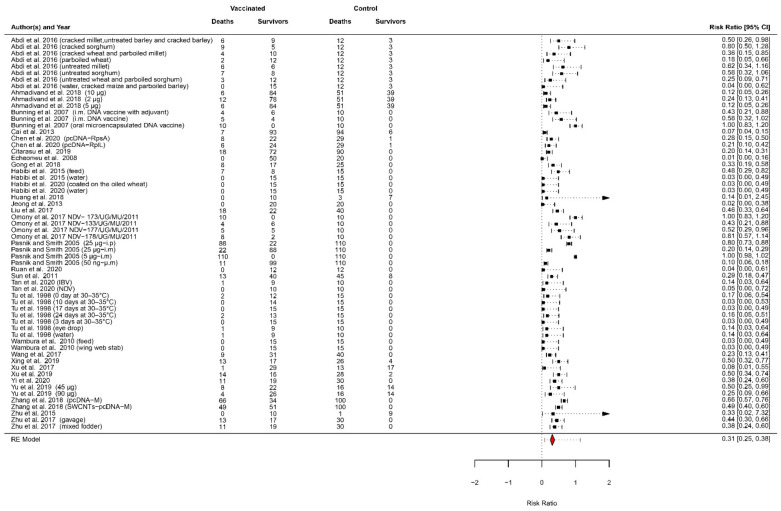
Forest plot of the risk ratio as a measure of vaccine efficacy (1-RR). Heterogeneity: I^2^ = 95 (95% CI: 93–98), tau^2^ = 0.44 (0.31–1.09), Q = 781.99, *p*-value < 0.0001.

The pooled RR was 0.31 (95% CI: 0.25–0.38), resulting in a vaccine efficacy (VE) of 69%. A vaccine efficacy of 69% indicates a 69% reduction in the death rate among the vaccinated groups. Effect estimates and confidence intervals are presented in the forest plot (Figure 3). 

**Figure A1:**
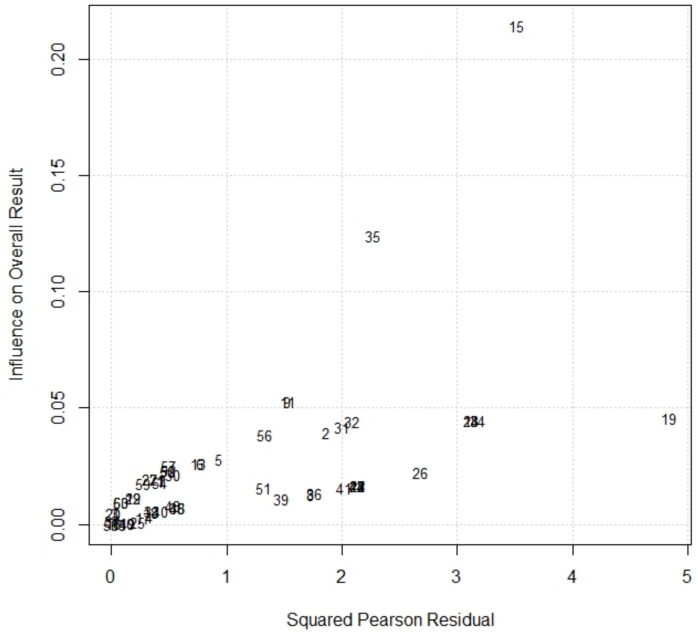
The Baujat Plot of roe deer studies. Study number 5 [90] could be an outlier which may distort the effect size estimate, as well as its precision.

**Figure A2:**
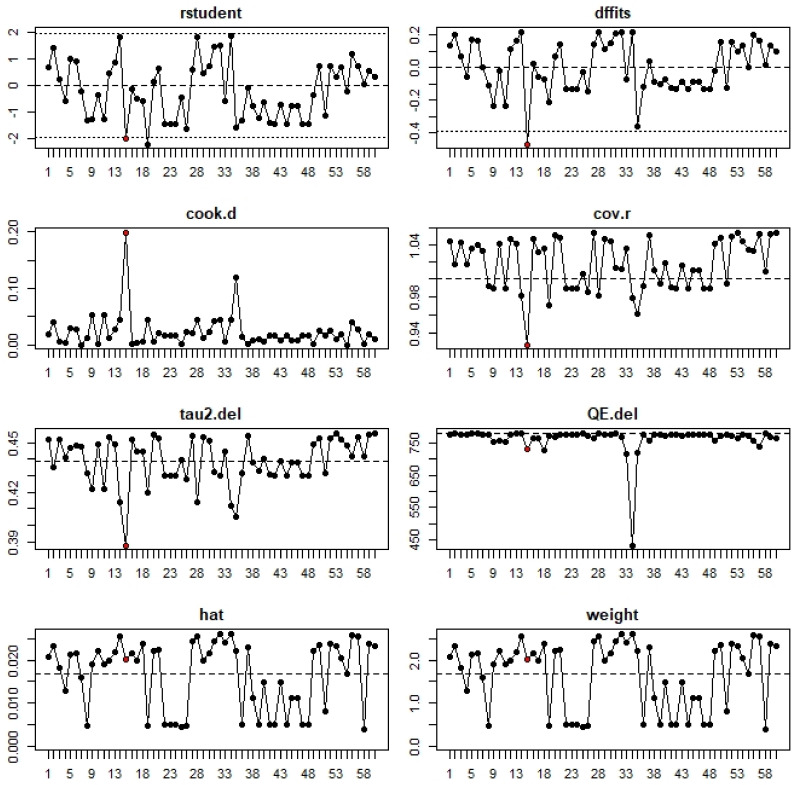
Influence analysis identifies study number 5 [90] as potential outlier.

**Figure A3:**
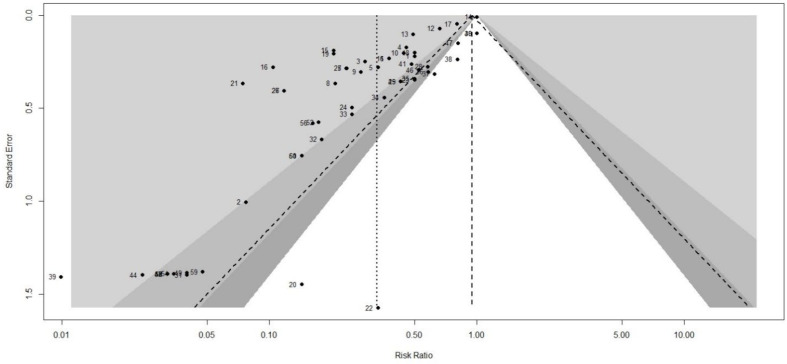
Funnel plot of the meta-analysis on vaccines efficacy.

The heterogeneity was significantly high, being I^2^ = 95 (95% CI: 93–98), with a *p*-value < 0.0001. One study was detected as influential, with an individual RR of 0.07 (93% of VE) (Appendix A: Figure A1 and Figure A2) [90]. Although the removal of this study would reduce the amount of heterogeneity and increase the precision of the estimated average outcome, we decided to keep it in the quantitative synthesis as it has one of the largest sample sizes (100 vaccinated animals and 100 control animals) and a high-quality study design. The inspection of the funnel plot shows some asymmetry (Appendix A: Figure A3). Nevertheless, the Peters’ test *p*-value was 0.27; therefore, the hypothesis of the symmetry of the funnel plot was accepted. A meta-analysis was not performed to evaluate the vaccine effectiveness due to the small number of field trials retrieved.

The subgroup analysis performed, according to the animal species, showed that the RRs were similar in fish (RR = 0.30 (95% CI: 0.22–0.40)) and avian species (RR = 0.31 (95% CI: 0.23–0.43)). Similar values were obtained for bacteria diseases (RR = 0.31 (95% CI: 0.21–0.44)) and virus diseases (RR = 0.30 (95% CI: 0.23–0.39)). For both moderators, the moderator test suggests that these variables do not influence the average VE (animal species: QM (df = 1) = 0.0585, *p*-value = 0.81; type of agent: QM (df = 1) = 0.0031, *p*-value = 0.95). Moreover, the test for residual heterogeneity significantly indicated that the other moderators not considered in the model were influencing the VE (animal species: I^2^ = 94 (95% CI: 92–98), *p*-value < 0.0001; type of agent: I^2^ = 94 (95% CI: 91–97) *p*-value < 0.0001).

## 4. Discussion

### 4.1. Summary of Evidence

This study represents the first systematic review and meta-analysis on the current state of thermostable vaccines against a selection of animal infectious diseases, providing a quantitative measure of their efficacy against death (VE = 69%). 

Most of the studies included are on vaccines against avian diseases, and, in particular, against NDV [42,43,44,45,46,52,53,54,55,56,57,58,67,69,70,71,73,75,76,78,79,82,83,84]. Developing a thermostable vaccine for Newcastle disease (ND) was considered a priority for non-governmental organisations (NGOs) and studies were funded to evaluate the effectiveness and economic viabilities of the vaccination in developing countries. Strong encouragement and support were provided by the FAO and the International Atomic Energy Agency (IAEA) to reduce the burden of the disease and improve the welfare of rural households [122,123]. In this context, the key success of the vaccination against NDV was the development of thermostable vaccines by the Australian Centre for International Agriculture Research (ACIAR) [124]. 

A similar situation can be observed for the vaccines developed against PPRV, for which progress has been driven by the PPR Global Control and Eradication Strategy (GCES) launched by the FAO and OIE [125]. The first thermostable vaccine (Nigeria 75/1 PPR strain) against this highly contagious disease has recently received the regulatory approvals required to be produced and commercially distributed in Nepal [126]. Along with Rinderpest, ND and PPR represent perfect examples of high-impact diseases which have benefitted from the support and incentives of NGOs by implementing a vaccination campaign with thermostable products.

Interestingly, and as a first step towards addressing the issue, some field studies provided robust evidence on the immunogenicity of commercial vaccines stored at temperatures far in excess of the manufacturer’s recommended cold-chain conditions [46,61]. Their results raise several questions, such as: (i) why manufacturers do not test for thermostability during vaccine development; (ii) why they do not include such information on the products labels; and (iii) how many other vaccines currently on the market could be stored outside the cold-chain, and for how long, whilst retaining equivalent potency? These studies provide preliminary evidence that some commercial vaccines might be used successfully, following a period of non-optimal storage in remote areas, regardless of the manufacturer’s recommendations. 

If we look back at recent and past history, our literature search highlights that the freeze-drying process is a valuable method to obtain vaccine thermostability [47,49,50,52,65,66,67,70,74,76,79,85]. An improved freeze-drying process was used to develop a thermostable Rinderpest vaccine (Thermovax), which was an essential tool for eradicating the disease in remote pastoral areas [127]. In this study we identified dried formulations (freeze- or foam-dried) for vaccines against NDV (e.g., [52]), bovine ephemeral fever virus (BEFV) [74], classical swine fever virus (CSFV) [85], rabies [77], and PPR [47], highlighting that the drying process is a useful technique to improve the thermostability of vaccines against diverse diseases in several species. However, it is worth mentioning that the drying process alone is not able to confer a long-term stability in the formulations. There are other ways that have been used to enhance the shelf life of the products at ambient temperatures. For instance, the freeze-dried vaccine against CSFV was stabilized with a buffer composed by trehalose, glycine, thiourea, and phosphate [85]. Other examples of stabilizers retrieved from this review include: (i) lactalbumin, hydrolysate, and sucrose for the Rinderpest vaccine [65] (ii) the methylglucoside for the vaccine against bovine ephemeral fever (BEF), (iii) and a formulation composed of trehalose, tryptone, and other protectants for the vaccine against the porcine reproductive and respiratory syndrome virus (PRRSV) [64]. 

Lyophilized vaccines are more stable prior to their reconstitution in the liquid form, while their potency is known to decline once reconstituted. In addition, not all vaccines can be lyophilized and, thus, there have been efforts to increase the stability of vaccines in liquid form. For instance, the stability of liquid vaccines can be achieved by optimizing the properties of the solvent (e.g., buffer, pH, and salt concentrations), and low-cost and safe excipients (e.g., glycerol) could provide freeze protection to vaccines with aluminum hydroxide, as an adjuvant to freeze damage [9]. Modern technologies have also become a key strategy to develop thermostable products. In this sense, Tan et al. [78] designed a thermostable recombinant NDV candidate vaccine against NDV and the infectious bronchitis virus (IBV), which was stable in the liquid form at 25 °C for 16 days. Similarly, Murr et al. [68] developed a recombinant NDV vector vaccine against PPR which was stable in the liquid form at −80 °C, −20 °C, 4 °C, 21 °C, and 37 °C for seven days.

Oral vaccinations are easy to implement and avoids stress in animals. Some thermostable vaccines have been developed with this route of administration in mind. The vaccine is incorporated into the feed during production, or it may be coated with pellets or encapsulated. Oral vaccines are particularly suitable for use in wild animals. In this sense, Smith et al. [77] developed a promising thermostable RABV vaccine using a foam drying process, highlighting the potential of this technique to produce a vaccine for oral use. The failure of the oral vaccination in the research by Bunning et al. [89] could have been due to the inactivation of the vaccines within the avian gastrointestinal tract. Oral vaccination was implemented in 16 other studies. These include articles on ND, using water and feed as vaccine carriers (e.g., [42,43,76,83]). The disadvantages of this route of administration are related to the large dose required to induce a uniform and long-lasting protection. For this reason, ND vaccines administered by eye-drops or treated feed have better performance than using water or untreated feed [42,43,57]. Additionally, oral vaccines may have an additional cost for the encapsulation, which may be necessary to avoid their degradation in the gastrointestinal environment prior to absorption [33].

Although a large number of trials using heat-stable vaccines was retrieved, very few peer-reviewed analyses exist on freeze-stable formulations. This finding shows how most efforts were directed to prevent vaccine deterioration and overcome the difficulty of maintaining the cold-chain in developing countries, which generally have high ambient temperatures. It is important to consider that although heat stability is perceived as a greater concern [128], conditions leading to freeze exposure occur, and may have an impact on the long-term stability of the vaccines, especially of those with aluminum adjuvants [9]. Damage due to freezing is likely in low- and middle-income countries, where cheap domestic refrigerators and cold boxes are used for storing and transporting vaccines. In particular, the poor performance of these refrigerators may lead to regular negative excursions, with potential damages to the vaccines during their storage [129]. Likewise, placing the vaccines with ice or gel packs inside portable containers may cause freeze damage to the vials too close to the ice and gel packs during their transportation [21]. With the exception of one article [68], this systematic review failed to identify studies in which the evaluation of the effect of freezing on vaccine potencies were assessed and, therefore, precluded identifying products fitting the definition of thermostable vaccines provided by the WHO [29]. Unsurprisingly, the information on heat stability and environmental temperatures, as provided by the authors, was reported heterogeneously in terms of different temperatures and periods of time (in ranges of days (e.g., Murr et al. [68]), weeks (e.g., Tu et al. [79]), or months (e.g., Dulal et al. [51]). Additionally, some authors defined the vaccines as thermostable only by performing a heat-treatment test in the lab (e.g., 56 °C for 60 min [58,73]). This diversity among benchmarks between the studies highlights the urgency to define standards when it comes to environmental or the freeze stability of vaccines. 

With regards to the search on DNA vaccines, most of the articles retrieved were on vaccines that were experimentally developed. Although many DNA vaccine candidates have been evaluated with promising results in various animal species, it has been estimated, by a recent review, that only five DNA vaccines have been approved and licensed for veterinary use [130]. These include:

• Three against viral diseases;
  ◦ Two for fish (one against infectious hematopoietic necrosis virus (IHNV), and one against salmon alphavirus subtype 3);
  ◦ One for horses against WNV, but used also in several avian species;
• One to treat cancer melanoma in dogs;
• One growth hormone-releasing hormone (GHRH) gene therapy for swine.

Conversely, no DNA vaccines have been licensed for human use to date [33,130]. DNA vaccination involves immunization with a plasmid encoding a gene of the pathogen. The production of DNA vaccines is cheaper than other types of vaccines. They are able to act in the presence of maternal antibodies, are temperature stable, and are safe to transport, which is especially important for remote areas [33]. Despite these advantages, some concerns have been raised, as DNA vaccines have failed to produce measurable antibodies, even if the host got protected, suggesting a major role of cellular immune responses. Another important concern is related to the potential deleterious effects following the integration into the host chromosome [131]. These issues, along with the cost of GMP (good manufacturing practices) grades, large-scale manufacturing restrains the commercial availability of DNA vaccines. 

In the majority of the articles on DNA vaccines, both humoral and cellular immune responses were assessed, obtaining promising results on the production of a variety of immune modulators, cytokines, and co-stimulatory molecules (e.g., [102]). DNA vaccines have received particular attention in the field of aquaculture. They are safe for fish since they do not contain an oil adjuvant that can cause peritonitis, but also for the consumer, as the fish are consumed months after vaccination and the quantity of DNA used is very small [33]. In this work, 16 out of 35 studies on DNA vaccines were carried out on fish species in China. Since China is a major player in global aquaculture, contributing to roughly 61% of the total production [132], it is not surprising that researchers from China conducted extensive research on DNA vaccines against different diseases impacting aquaculture. In fish, the RPS, post-challenge, in the groups vaccinated ranged from 20% for the vaccine against *Mycobacterium marinum* developed by Pasnik and Smith [105], to 92% for the vaccine against *Vibrio alginolyticus* developed by Cai et al. [90] and the vaccine against IHNV and the infectious pancreatic necrosis virus (IPNV) developed by Xu et al. [113]. It should also be considered that the immune efficiency varies based on the immunization routes, doses, and times of DNA immunization. In fact, Pasnik and Smith [105] reported a higher protection (RPS: 80–90%) for the same vaccine administered at a higher dose, and a lower RPS at lower vaccine dose (RPS: 0%). Our search also retrieved a great number of studies on DNA vaccines against avian diseases. Promising results have been obtained in avian species, with an RPS, post-challenge, ranging from 44% for the vaccine against WNV in the American crow [89] to 100% for the vaccine against novel duck reovirus (NDR) in ducks [120]. However, Bunning et al. [89] showed that the response to the DNA vaccines depended on the inclusion of an adjuvant (RPS: 60%) and the route of administration, as none of the birds receiving the oral microencapsulated DNA vaccine against WNV developed antibodies, and none of them survived post-challenge (RPS: 0%). 

The VE, in terms of protection against death, is an objective measure to aggregate data on different vaccines. Indeed, numbers or rates of death are the most used measure for comparing the impact of different diseases in epidemiology [133]. In vaccine trials, challenging humans with dangerous pathogens is ethically unacceptable. Conversely, the evaluation of veterinary vaccines mainly relies on challenge studies. This is important to consider as serological studies may not always provide a good measure of efficacy [134]. For all these reasons, the pooled estimate of the VE was provided in terms of the reduced risk of death. The protection of thermostable vaccines against fatal diseases was estimated to be high (VE = 69%), highlighting the benefits of vaccination to reduce the economic losses (direct deaths) due to the disease impact. The heterogeneity between studies was high. 

Developing a portfolio of thermostable vaccines would not only help with improving access to vaccines in parts of the world where cold-chain capacity is lacking, overcoming a major supply-chain hurdle to the rollout of successful vaccination campaigns for humans and animals, but it would also greatly benefit the environment by reducing the great consumption of energy required to sustain the cold-chain. On top of the overall energy consumption of an increased number of refrigeration units, maintaining ultracold temperatures requires the use of hydrofluorocarbon gases, which are known to have a very heavy carbon footprint [135]. An additional benefit can be obtained by investing in thermostable products that can aid eradication programs, such as “differentiating infected from vaccinated animals” DIVA-vaccines, such as the ones presented in this review, developed by Verardi et al. [80], Daouam et al. [49], Dulal et al. [51], and Murr et al. [68]. These types of vaccines are promising for the effective disease control during outbreaks, and eradication programs in disease-endemic regions [136].

### 4.2. Limitations

The current study should be interpreted within the context of its limitations. Firstly, it does not provide a complete overview of the licensed thermostable vaccines for veterinary medicine. Instead, it aims to synthetize the peer-reviewed articles on thermostable vaccines developed against a selection of animal diseases. The target is not only to include the commercial vaccines, but also the vaccines experimentally developed, which are promising candidates. Additionally, only studies testing for VE, and the protection of the target hosts, were included, while in vitro studies, or studies testing the vaccines on non-natural hosts, were excluded. The intent was to retrieve an adequate number of studies to summarize the evidence on the efficacy of thermostable vaccines, rather than describe the progress made in vaccine technology. Some successful technologies that produce vaccine thermostability may not have been included in this study because the peer-reviewed articles were on vaccines tested under laboratory conditions.

Secondly, the search on DNA vaccines was intentionally limited by sorting for relevance and extracting the first 100 records from each bibliographic database. The screening of all the papers would have allowed us to retrieve a higher number of articles, which would have compromised the time efficiency of our search. Indeed, such an approach would have been unfeasible, given the growing number of peer-reviewed articles on DNA vaccine candidates for animal species [137]. Moreover, in this case, the aim was not to provide a comprehensive overview on DNA vaccines for veterinary use, which has been reviewed by several narrative reviews (e.g., Fomsgaard and Liu [130]). Instead, this study aims to highlight some applications of these vaccines, which have intrinsic thermostability characteristics.

Thirdly, considering that the methodology to assess the immunogenicity, durability of immunity, and the safety profile is specific to each disease, comparisons on the humoral and cell-mediated immunities elicited by the vaccines were not made. The outcomes of interest for veterinary vaccines consider the livestock profitability and vary according to the disease. In the articles extracted, the outcomes ranged from the evaluation of specific disease symptoms (in cases of non-acute diseases) (e.g., Murr et al. [68]) to mortality. These different conditions could not have been compared or pooled. 

Fourthly, the heterogeneity of the included studies is likely to be due to the different diseases against which the vaccines have been developed. Because of the small number of articles for each disease, the heterogeneity was not investigated using a subgroup analysis according to the disease. Additionally, other factors influencing the performance of vaccines were not assessed. These include the age and sex of the animals, the level and time of the challenge (pathogen factors), the dose, and the route of vaccine administration. Despite these limitations, it is important to consider that most of the individual estimates show the same direction of effect (RR < 1), highlighting the significant protection conferred by the vaccination.

## 5. Conclusions

This study presents the first condensed evidence from peer-reviewed literature on the current availability of thermostable vaccines for veterinary use. Over the years diverse methods have been implemented to develop and improve vaccine thermostability. Moreover, the efficacy of these formulations has been proved for several animal diseases, with an overall risk of death, in vaccinated animals, that is reduced by nearly 70% compared with unvaccinated controls. Although we were not able to identify the exact percentage of thermostable formulations, many articles cited in this review stated that most of vaccines on the market are still dependent on cold-chain systems, stressing the importance of enhancing their stability (e.g., [9,18,127]). The recent COVID-19 pandemic has highlighted the difficulties in transporting and storing non-thermostable vaccine formulations, especially for low-income countries, highlighting the necessity to improve the distribution and storage of vaccines to adequately respond to the current and future pandemics. In this regard, the reevaluation of vaccine research and development, manufacturing, and supply-chain management strategies are essential to produce vaccines that are heat- and freeze-stable to make vaccinations widely available to anyone globally, regardless of cold-chain capacity. We suggest that each novel vaccine candidate should be evaluated for its thermostability along with its safety, immunogenicity, and protective efficacy before it is licensed for use. The shelf life of existing products should be investigated, by default, under non-cold-chain conditions, coupled with efforts to boost their thermostability. We also strongly encourage regulatory agencies to adopt a standard definition of vaccine heat- and freeze-stability requirements to be used for the development of new generation vaccines both for human and for veterinary use.

As a final point, we would like to invite funding agencies and donors who support vaccine research to reflect and consider on the added value that having more stable products would bring to their philanthropic efforts both in human and veterinary medicine.

## Figures and Tables

**Table 1:** Computerized literature search using database-appropriate syntax.

Database	Strategy	No. of Publications
Thermostable Vaccines		
PubMed	(“vaccin*”[Title/Abstract] AND (“thermostable”[Title/Abstract] OR “heat stable”[Title/Abstract] OR “freeze stable”[Title/Abstract] OR (“heat-freeze”[All Fields] AND “stable”[Title/Abstract]))) AND ((fft[Filter]) AND (1990:2021[pdat]))	462
CAB Abstracts	(title:(vaccin*) OR ab:(vaccin*))AND (title:(thermostable) OR ab:(thermostable) OR title:(heat stable) OR ab:(heat stable) OR title:(freeze stable) OR ab:(freeze stable) OR title:(heat-freeze stable) OR ab:(heat-freeze stable)) AND yr:[1990 TO 2021]	500
Web of Science	(TI = (vaccin*) OR AB = (vaccin*)) AND (TI = (thermostable) OR AB = (thermostable) OR TI = (heat stable) OR AB = (heat stable) OR TI = (freeze stable) OR AB = (freeze stable) OR TI = (heat-freeze stable) OR AB = (heat-freeze stable)) Timespan: 1 January 1990 to 5 September 2021 (Publication Date) Not: Document Types: Proceedings Papers or Editorial Materials or Meeting Abstracts or Book chapters or Notes or Early access	693
DNA Vaccines		
PubMed	(“vaccines, dna”[MeSH Major Topic] AND “animals”[MeSH Major Topic]) AND ((fft[Filter]) AND (english[Filter]))	417 First 100 sorted by best match
CAB Abstracts	title:(DNA vaccine) OR ab:(DNA vaccine) AND up:(Animals) AND yr:[1996 TO 2021] Refinements: Document type = Journal article AND Language = English	6845 First 100 sorted by relevance
Web of Science	(TS = (“DNA vaccine”)) AND (DT == (“ARTICLE”) AND TASCA == (“VETERINARY SCIENCES”) AND LA == (“ENGLISH”))	557 First 100 sorted by relevance

**Table 2:** Overview of the studies included in the qualitative synthesis retrieved from the broad search on thermostable vaccines.

Study	Target Agent	Type of Agent	Animal Species	Country	Product Name	Vaccine Type	Strain/Gene	Market Availability	Thermostability Characteristics	Route of Administration	Type of Study	Assessment
Abah et al. [42]	Newcastle disease virus	Virus	Chicken	Nigeria	N.A.	Live-attenuated	I-2	Locally produced	N.A.	Oral (feed)	Clinical trial (vaccinated vs. control)	Assessment of humoral immunity
Abdi et al. [43]	Newcastle disease virus	Virus	Chicken	Ethiopia	NDV vaccine (National Veterinary Institute of Bishoftu, Ethiopia)	Live-attenuated ^2^	I-2	Commercially available	N.A.	Oral (feed and water)	Clinical trial (vaccinated vs. control)	Assessment of humoral immunity, clinical signs, and RPS post-challenge
Acharya et al. [44]	Newcastle disease virus	Virus	Chicken	Nepal	N.A.	Live-attenuated	I-2	Locally produced	30 °C for 7 days	Intraocular	Field trial (all animals vaccinated)	Assessment of humoral immunity
Asl Najjari et al. [45]	Newcastle disease virus	Virus	Chicken	Iran	N.A.	Live-attenuated	I-2	Locally produced	N.A.	Intraocular	Clinical trial (vaccinated vs. control)	Assessment of humoral immunity, clinical signs, and RPS post-challenge
Awa et al. [46]	Newcastle disease virus	Virus	Chicken	Cameroon	Multivax (LANAVET Garoua, Cameroon)	Live-attenuated	La Sota + Cholevax + Typhovax	Commercially available	34 °C for 10 weeks	Intramuscolar	Clinical and field trials (all animals vaccinated)	Assessment of humoral immunity
Balamurugan et al. 2014 [47]	Peste des petits ruminants virus	Virus	Goats	India	N.A.	Live-attenuated ^3^	Jhansi/2003	Experimentally developed	24–26 days at 25 °C 7–8 days at 37 °C 3–4 days at 40 °C (Riyesh et al. [48])	Subcutaneous	Clinical trial (vaccinated vs. control)	Assessment of humoral immunity, clinical signs, and RPS post-challenge
Daouam et al. [49]	Rift Valley Fever virus	Virus	Cattle, sheep, and goats	Morocco	N.A.	Live-attenuated ^2^	Clone of CL13T	Experimentally developed	37 °C for 4 days 20 months at 4 °C	Subcutaneous	Clinical trial (vaccinated vs. control)	Assessment of humoral immunity
Daouam et al. [50]	Rift Valley Fever virus	Virus	Camels	Morocco	N.A.	Live-attenuated ^2^	Clone of CL13T	Experimentally developed	(see Daouam et al. [49])	Subcutaneous	Clinical trial (all animals vaccinated)	Assessment of humoral immunity
Dulal et al. [51]	Rift Valley Fever virus	Virus	Cattle	United Kingdom	ChAdOx1-GnGc	Recombinant vector ^4^	MP-12	Experimentally developed	25°, 37°or 45 °C for 6 months	Intramuscolar	Clinical trial (all animals vaccinated)	Assessment of humoral immunity
Echeonwu et al. [52]	Newcastle disease virus	Virus	Chicken	Nigeria	N.A.	Live-attenuated ^2^	I-2	Locally produced	N.A.	Oral (feed)	Clinical trial (vaccinated vs. control)	Assessment of humoral immunity, clinical signs, and RPS post-challenge
Foster et al. [53]	Newcastle disease virus	Virus	Chicken	Tanzania	Websters HR V4	Live-attenuated	V-4	Locally produced	N.A.	Intraocular and oral (water)	Field trial (vaccinated vs. control)	Assessment of humoral immunity
Habibi et al. [54]	Newcastle disease virus	Virus	Chicken	Iran	N.A.	Live-attenuated	I-2	Locally produced	N.A.	Oral (feed and water)	Clinical trial (vaccinated vs. control)	Assessment of humoral immunity, clinical signs, and RPS post-challenge
Habibi et al. [55]	Newcastle disease virus	Virus	Chicken	Iran	N.A.	Live-attenuated	I-2	Locally produced	N.A.	Oral (feed)	Clinical trial (vaccinated vs. control)	Assessment of humoral immunity, clinical signs, and RPS post-challenge
Henning et al. [56]	Newcastle disease virus	Virus	Chicken	Myanmar	N.A.	Live-attenuated	I-2	Locally produced	N.A.	Intraocular and intranasal	Field trial (vaccinated vs. control)	Assessment of humoral immunity
Illango et al. [57]	Newcastle disease virus	Virus	Chicken	Uganda	N.A.	Live-attenuated	I-2	Locally produced	N.A.	Oral (water)	Clinical trial (vaccinated vs. control)	Assessment of humoral immunity
Jeong et al. [58]	Newcastle disease virus	Virus	Chicken	Korea	N.A.	Live-attenuated	K148/08	Experimentally developed	Thermostability test ^13^	Cabinet sprayer and Intraocular	Clinical trial (vaccinated vs. control)	Assessment of Humoral immunity, histopathological lesions, and RPS post-challenge
Jones et al. [59]	Peste des petits ruminants virus	Virus	Goats	USA	vRVFH	Recombinant vector ^5^	F and H (Rinderpest)	Experimentally developed	N.A.	Intramuscolar	Clinical trial (vaccinated vs. control)	Assessment of humoral immunity and clinical signs post-challenge
Khandelwal et al. [60]	Peste des petits ruminants virus	Virus	Sheep	India	N.A.	Recombinant subunit ^6^	HN	Experimentally developed	N.A.	Oral (feed)	Field trial (all animals vaccinated)	Assessment of humoral immunity
Lankester et al. [61]	Rabies	Virus	Dogs	Tanzania	Nobivac (rabies, MSD Animal Health, Boxmeer, The Netherlands)	Live-attenuated	Pasteur RIV	Commercially available	25 °C for 6 months and 30 °C for 3 months	Subcutaneous	Field trial (all animals vaccinated)	Assessment of humoral immunity
Liu et al. [62]	*Mycoplasma gallisepticum*	Bacterium	Chicken	China	N.A.	Recombinant vector ^7^	pmga1.2p	Experimentally developed	N.A.	Intra-gastric gavage	Clinical trial (vaccinated vs. control)	Assessment of humoral immunity
Liu et al. [63]	Avian influenza virus	Virus	Chicken	China	N.A.	Recombinant vector ^7^	NP of H9N2	Experimentally developed	N.A.	Intra-gastric gavage	Clinical trial (vaccinated vs. control)	Assessment of humoral immunity
Lv et al. [64]	Porcine reproductive and respiratory syndrome virus	Virus	Pigs	China	N.A.	Live-attenuated ^8^	JXA1-R	Experimentally developed	25 °C for 12 months and 37 °C for 4 months	Not specified	Clinical trial (vaccinated vs. control)	Assessment of humoral immunity
Mariner et al. [65]	Rinderpest virus	Virus	Cattle	Nigeria	VRPV	Live-attenuated ^3^	RBOK	Experimentally developed	25.9 °C (17.1–37.8 °C) for 34 days	Subcutaneous	Field trial (vaccinated vs. control)	Assessment of humoral immunity
Mariner et al. [66]	Peste des petits ruminants virus	Virus	Goats	USA	TVRPV	Live-attenuated ^3^	RBOK	Experimentally developed	37 °C for up to 245 days	Subcutaneous	Clinical trial (vaccinated vs. control)	Assessment of humoral immunity, clinical signs, and RPS post-challenge
Mehrabadi et al. [67]	Newcastle disease virus	Virus	Chicken	Iran	ND.TR.IR (Razi Institute, Iran)	Live-attenuated ^2^	I-2	Commercially available	N.A.	Oral (water)	Field trial (vaccinated vs. control)	Assessment of humoral immunity
Murr et al. [68]	Peste des petits ruminants virus	Virus	Goats	Germany	rNDV_HKur	Recombinant vector ^9^	Kurdistan/11/H	Experimentally developed	−80 °C, −20 °C, 4 °C, 21 °C, and 37 °C for 7 days	Subcutaneous	Clinical trial (vaccinated vs. control)	Assessment of humoral immunity and clinical signs post-challenge
Nega et al. [69]	Newcastle disease virus	Virus	Chicken	Ethiopia	N.A.	Live-attenuated	I-2	Locally produced	N.A.	Intraocular	Field trial (all animals vaccinated)	Assessment of humoral immunity, clinical signs, and RPS post-challenge
Nwanta et al. [70]	Newcastle disease virus	Virus	Chicken	Nigeria	NDV4HR (Malaysian Vaccines and Pharmaceutical SNP BHD, Malaysia)	Live-attenuated ^2^	V-4	Commercially available	N.A.	Oral (feed)	Field trial (all animals vaccinated)	Assessment of humoral immunity
Omony et al. [71]	Newcastle disease virus	Virus	Chicken	Uganda	N.A.	Live-attenuated	NDV-133/UG/MU/2011, NDV-177/UG/MU/2011NDV-178/UG/MU/2011 and NDV-173/UG/MU/2011	Experimentally developed	N.A.	Intraocular and Intranasal	Clinical trial (vaccinated vs. control)	Assessment of humoral immunity, clinical signs, and RPS post-challenge
Rahman et al. [72]	Peste des petits ruminants virus	Virus	Goats	Bangladesh	N.A.	Live-attenuated	N.A.	Experimentally developed	Percent inhibition values decreased by 8–20% at 180 DPV, when the vaccine is kept 25°, 30°, 35°, and 40 °C for 7 and 14 days	Subcutaneous	Clinical trial (vaccinated vs. control)	Assessment of humoral immunity
Ruan et al. [73]	Newcastle disease virus	Virus	Chicken	China	N.A.	Live-attenuated ^10^	rHR09	Experimentally developed	Thermostability test ^13^	Intramuscolar	Clinical trial (vaccinated vs. control)	Assessment of humoral immunity, clinical signs, and RPS post-challenge
Shendy et al. [74]	Bovine ephemeral fever virus	Virus	Cattle	Egypt	N.A.	Live-attenuated ^2^	BEF/AVS/2000	Experimentally developed	25 °C for 6 months 37 °C for 3 months 45 °C for 20 days	Subcutaneous	Clinical trial (vaccinated vs. control)	Assessment of humoral immunity
Siddique et al. [75]	Newcastle disease virus	Virus	Chicken	Pakistan	N.A.	Live-attenuated	I-2	Locally produced	N.A.	Oral (water)	Clinical trial (vaccinated vs. control)	Assessment of humoral immunity
Siddique et al. [76]	Newcastle disease virus	Virus	Ring-necked pheasants	Pakistan	N.A.	Live-attenuated ^2^	I-2	Locally produced	28 °C for 6–8 weeks and 4–8 °C for 1 year	Oral (feed)	Field trial (vaccinated vs. control)	Assessment of humoral immunity
Smith et al. [77]	Rabies virus	Virus	Gray foxes	USA	N.A.	Live-attenuated ^8^	ERA	Experimentally developed	22° ± 4 °C for up to 65 days	Intestinal endoscopy	Clinical trial (vaccinated vs. control)	Assessment of humoral immunity
Tan et al. [78]	Newcastle disease virus and infectious bronchitis virus	Virus	Chicken	China	rLS-T-HN-T/B	Recombinant bivalent live ^12^	HN and S1	Experimentally developed	25 °C for 16 days	Intraocular and intranasal	Clinical trial (vaccinated vs. control)	Assessment of humoral immunity, clinical signs, and RPS post-challenge
Tu et al. [79]	Newcastle disease virus	Virus	Chicken	Vietnam	N.A.	Live-attenuated ^2^	I-2	Locally produced	30 °C for 3 weeks	Intraocular and oral (water)	Clinical and field trials (vaccinated vs. control)	Assessment of humoral immunity, clinical signs, and RPS post-challenge
Verardi et al. [80]	Rinderpest virus	Virus	Cattle	Ethiopia and Kenya	N.A.	Recombinant vector ^11^	v2RVFH	Experimentally developed	N.A.	Intramuscolar	Field trial (vaccinated vs. control)	Assessment of humoral immunity, clinical signs, and RPS post-challenge
Wambura et al. [81]	Flow pox virus	Virus	Chicken	Tanzania	N.A.	Live-attenuated	TPV-1	Locally produced	25–34 °C for 6 months	Wing web stab	Clinical trial (vaccinated vs. control)	Assessment of humoral immunity, clinical signs, and RPS post-challenge
Wambura et al. [82]	Newcastle disease virus	Virus	Chicken	Tanzania	N.A.	Live-attenuated	I-2	Locally produced	N.A.	Oral (feed and water) and ocular	Clinical trial (vaccinated vs. control)	Assessment of humoral immunity, clinical signs, and RPS post-challenge
Wambura et al. [83]	Newcastle disease virus	Virus	Helmeted guinea fowls	Tanzania	N.A.	Live-attenuated	I-2	Locally produced	N.A.	Oral (feed)	Field trial (vaccinated vs. control)	Assessment of humoral immunity, clinical signs, and RPS post-challenge
Wen et al. [84]	Newcastle disease virus	Virus	Chicken ^1^	China	N.A.	Live-attenuated	TS09-C	Experimentally developed	N.A.	In ovo	Clinical trial (vaccinated vs. control)	Assessment of humoral immunity, histopathological lesions, and RPS post-challenge
Zuo et al. [85]	Classical swine fever virus	Virus	Pigs	China	ST16	Live-attenuated ^2^	C	Experimentally developed	25 °C for 6 months	Intramuscolar	Clinical trial (vaccinated vs. control)	Assessment of humoral immunity

^1^ SPF chicken embryos, ^2^ Freeze-dried, ^3^ Freeze-dried Vero cell-adapted, ^4^ Chimpanzee adenovirus vector, ^5^ Double recombinant Vaccina virus, ^6^ Transgenic peanut, ^7^ *Bacillus thurigensis* vector, ^8^ Foam-dried, ^9^ NDV vector, ^10^ Generated by reverse genetics system, ^11^ Vaccina virus vector, ^12^ NDV vector, ^13^ Thermostability test according to Wen et al. [86].

**Table 3:** Overview of the studies included in the qualitative synthesis retrieved from the search on DNA vaccines.

Study	Target Agent	Type of Agent	Animal Species	Country	Product Name	Encoding Gene	Market Availability	Route of Administration	Type of Study	Assessment
Ahmadivand et al. [87]	Infectious pancreatic necrosis virus	Virus	Fish (rainbow trout)	Iran	pcDNA3.1-VP2	VP2	Experimentally developed	Intramuscular	Clinical trial (vaccinated vs. control)	Assessment of humoral and cell-mediated immunity, clinical signs, and survival rate post-challenge
Bande et al. [88]	Avian infectious bronchitis coronavirus	Virus	Chicken	Malaysia	pBudCR88-S1/M41-S1	S1 glycoprotein	Experimentally developed	Intramuscular	Clinical trial (vaccinated vs. control)	Assessment of humoral and cell-mediated immunity, and histopathological lesions post-challenge
Bunning et al. [89]	West Nile virus	Virus	American crows	USA	N.A.	prM and E	Experimentally developed	Oral and intramuscular	Clinical trial (vaccinated vs. control)	Assessment of humoral immunity, clinical signs, and survival rate post-challenge
Cai et al. [90]	*Vibrio alginolyticus*	Bacterium	Fish (crimson snapper)	China	pcDNA-ompW	ompW	Experimentally developed	Intramuscular	Clinical trial (vaccinated vs. control)	Assessment of humoral immunity, clinical signs, and survival rate post-challenge
Chen et al. [91]	*Nocardia seriolae*	Bacterium	Fish(hybrid snakehead)	China	pcDNA-RplL and pcDNA-RpsA	RpsA and RplL	Experimentally developed	Intramuscular	Clinical trial (vaccinated vs. control)	Assessment of humoral and cell-mediated immunity, clinical signs, and survival rate post-challenge
Citarasu et al. [92]	Macrobrachium rosenbergii nodavirus	Virus	Fish(giant freshwater)	India	MrNV-CP-RNA-2-pVAX1	MrNV-CP-RNA-2	Experimentally developed	Oral (feed)	Clinical trial (vaccinated vs. control)	Assessment of immunological and hematological parameters, and survival rate post-challenge
Clapp et al. [93]	*Brucella abortus*	Bacterium	Bison	USA	pCMVbp26 + pCMVTF	bp26 + TF	Experimentally developed	N.A.	Clinical trial (vaccinated vs. control)	Assessment of humoral and cell-mediated immunity
Cui et al. [94]	Porcine reproductive and respiratory syndrome virus	Virus	Pigs	USA	DNA GP5-Mosaic/VACV GP5-Mosaic	ATCC VR-2332 and MN184C	Experimentally developed	Intradermal and intramuscular	Clinical trial (vaccinated vs. control)	Assessment of humoral and cell-mediated immunity, clinical signs, and survival rate post-challenge
Dahiya et al. [95]	Canine parvovirus	Virus	Dogs	India	pAlpha-CPV-VP2	VP2	Experimentally developed	Intradermal	Clinical trial (vaccinated vs. control)	Assessment of humoral and cell-mediated immunity
Davis et al. [96]	West Nile virus	Virus	Penguins	USA	WNDV Vaccine (Aldevron Llc, Fargo, North Dakota, USA).	prM/M and E	Commercially available	Intramuscular	Clinical trial (vaccinated vs. control)	Assessment of humoral immunity
Eman et al. [97]	Avian influenza(H5N1)	Virus	Chicken	India	pDEST 40/H5 and pDEST 40/N1	H5 and N1	Experimentally developed	Ocular	Clinical trial (vaccinated vs. control)	Assessment of humoral immunity
Fu et al. [98]	Duck hepatitis virus type 1	Virus	Ducks	China	pSCA/VP1	VP1	Experimentally developed	Intramuscular	Clinical trial (vaccinated vs. control)	Assessment of humoral and cell-mediated immunity, clinical signs, and survival rate post-challenge
Garver et al. [99]	Infectious hematopoietic necrosis virus	Virus	Fish(spring chinook, sockeye and kokanee salmon fry)	USA	pIHNw-G	G	Experimentally developed	Intramuscular	Clinical trial (vaccinated vs. control)	Assessment of humoral immunity
Gong et al. [100]	*Pasteurella multocida*	Bacterium	Chicken	China	N.A.	ptfA	Experimentally developed	Intramuscular	Clinical trial (vaccinated vs. control)	Assessment of humoral and cell-mediated immunity, clinical signs, and survival rate post-challenge
Huang et al. [101]	Duck Tembusu Virus	Virus	Ducks	China	pVAX1-C	Capsid gene (GenBank: JX196334.1)	Experimentally developed	Oral	Clinical trial (vaccinated vs. control)	Assessment of humoral immunity, clinical signs, and survival rate post-challenge
Kotla et al. [102]	Foot-and-mouth disease virus	Virus	Cattle	India	P1-2A-3CpCDNA + bIL-18pCDNA	P1-2A-3C + bovine IL-18	Experimentally developed	Intramuscular	Clinical trial (vaccinated vs. control)	Assessment of humoral and cell-mediated immunity
Liu et al. [103]	*Edwardsiella tarda*	Bacterium	Fish (olive flounder)	China	pCG-OmpC	OmpC	Experimentally developed	Intramuscular	Clinical trial (vaccinated vs. control)	Assessment of humoral and cell-mediated immunity, clinical signs, and survival rate post-challenge
Liu et al. [104]	*Campylobacter* spp.	Bacterium	Chicken	USA	pCAGGS_CfrA and pCAGGS_CmeC	cfrA and cmeC	Experimentally developed	In ovo	Clinical trial (all animals vaccinated)	Assessment of humoral and intestinal colonization post-challenge
Pasnik and Smith [105]	*Mycobacterium marinum*	Bacterium	Fish(hybrid striped bass)	USA	pCMV-85A	Ag85A	Experimentally developed	Intramuscular	Clinical trial (vaccinated vs. control)	Assessment of humoral and cell-mediated immunity, clinical signs, and survival rate post-challenge
Sisteré-Oró et al. [106]	Swine influenza virus	Virus	Pigs	Spain	VC4-flagellin DNA	VC-4-flagel-lin (constructed multipeptide)	Experimentally developed	Intradermal (IDAL1 device, MSD Animal Health)	Clinical trial (vaccinated vs. control)	Assessment of humoral and cell-mediated immunity, histopathological lesions, and survival rate post-challenge
Sun et al. [107]	*Edwardsiella tarda*	Bacterium	Fish (olive flounder)	China	pCEsa1	Esa1	Experimentally developed	Intramuscular	Clinical trial (vaccinated vs. control)	Assessment of humoral and cell-mediated immunity, histopathological lesions, and survival rate post-challenge
Tarradas et al. [108]	Classical swine fever virus	Virus	Pigs	Spain	pE2 and pCCL20	E2 and swine CCL20	Experimentally developed	Intramuscular	Clinical trial (vaccinated vs. control)	Assessment of humoral and cell-mediated immunity, and clinical signs, post-challenge
Vaughan et al. [109]	Dolphin morbillivirus	Virus	Atlantic bottlenose dolphins	USA	pVR-DMV-F and pVR-DMV-H (vaccinated)	Fusion (F) and hemagglutinin (H)	Experimentally developed	Intramuscular	Clinical trial (vaccinated vs. control)	Assessment of humoral and cell-mediated immunity
Wang et al. [110]	*Vibrio harvey*	Bacterium	Fish(orange-spotted grouper)	China	pcDNA-GPx	GPx	Experimentally developed	Intramuscular	Clinical trial (vaccinated vs. control)	Assessment of humoral immunity, clinical signs, and survival rate post-challenge
Wium et al. [111]	*Mycoplasma* spp.	Bacterium	Ostriches	South Africa	pCI-neo_oppA and VR1020_oppA	oppA	Experimentally developed	Intramuscular	Field trial (vaccinated vs. control)	Assessment of humoral immunity
Xing et al. [112]	*Vibrio anguillarum*	Bacterium	Fish (olive flounder)	China	pcDNA3.1-VAA (pVAA)	VAA	Experimentally developed	Intramuscular	Clinical trial (vaccinated vs. control)	Assessment of humoral and cell-mediated immunity, histopathological lesions, and survival rate post-challenge
Xu et al. [113]	Infectious hematopoietic necrosis virus and infectious pancreatic necrosis virus	Virus	Fish (rainbow trout)	China	pCh-IHN/IPN	G gene of IHNV Sn1203 and VP2 and VP3 genes of IPNV ChRtm213	Experimentally developed	Intramuscular	Clinical trial (vaccinated vs. control)	Assessment of humoral and cell-mediated immunity, histopathological lesions, and survival rate post-challenge
Xu et al. [114]	*Vibrio anguillarum*	Bacterium	Fish (olive flounder)	China	pcDNA3.1-OmpK (pOmpK)	OmpK	Experimentally developed	Intramuscular	Clinical trial (vaccinated vs. control)	Assessment of humoral and cell-mediated immunity, clinical signs, and survival rate post-challenge
Yang et al. [115]	Infectious bronchitis virus	Virus	Chicken	China	pVAX1-S1/M/N	S1,N,M	Experimentally developed	Intramuscular	Clinical trial (vaccinated vs. control)	Assessment of humoral and cell-mediated immunity, and clinical signs, post-challenge
Yi et al. [116]	Largemouth bass virus	Virus	Fish (largemouth bass)	China	pCDNA3.1(+)-MCP-Flag	MCP	Experimentally developed	Intramuscular	Clinical trial (vaccinated vs. control)	Assessment of humoral and cell-mediated immunity, clinical signs, and survival rate post-challenge
Yu et al. [117]	Singapore grouper iridovirus	Virus	Fish(grouper)	China	pcDNA3.1–19R	SGIV-19R	Experimentally developed	Intramuscular	Clinical trial (vaccinated vs. control)	Assessment of humoral and cell-mediated immunity, clinical signs, and survival rate post-challenge
Yuan et al. [118]	Rabbit hemorrhagic disease virus	Virus	Rabbits	China	pcDNA-VP60	VP60	Experimentally developed	Intramuscular	Clinical trial (vaccinated vs. control)	Assessment of humoral and cell-mediated immunity
Zhang et al. [119]	Spring viremia of carp virus	Virus	Fish(common carp)	China	pcDNA-M and SWCNTs-pcDNA-M	M	Experimentally developed	Intramuscular	Clinical trial (vaccinated vs. control)	Assessment of humoral and cell-mediated immunity, clinical signs, and survival rate post-challenge
Zhu et al. [120]	Novel duck reovirus	Virus	Ducks	China	pSCA/sigma C	Sigma C	Experimentally developed	Intramuscular	Clinical trial (vaccinated vs. control)	Assessment of humoral and cell-mediated immunity, histopathological lesions, and survival rate post-challenge
Zhu et al. [121]	*Streptococcus agalactiae*	Bacterium	Fish (Nile tilapia)	China	SL7207-pVAX1-sip	Sip	Experimentally developed	Oral (gavage and mixed fodder)	Clinical trial (vaccinated vs. control)	Assessment of humoral and cell-mediated immunity, clinical signs, and survival rate post-challenge

**Table 4:** Overview of the studies included in the quantitative synthesis to assess the pooled vaccine efficacies.

Study	Vaccinated Group Deaths	Vaccinated Group Survivals	Control Group Deaths	Control Group Survivals	Challenge Time (dpv)	Relative Percent of Survival (RPS)-Days Post-Challenge
Abdi et al. [43] (cracked millet, untreated barley, and cracked barley)	6	9	12	3	21	50%-28 days
Abdi et al. [43] (cracked sorghum)	9	5	12	3	21	20%-28 days
Abdi et al. [43] (cracked wheat and parboiled millet)	4	10	12	3	21	64%-28 days
Abdi et al. [43] (parboiled wheat)	2	12	12	3	21	82%-28 days
Abdi et al. [43] (untreated millet)	6	6	12	3	21	38%-28 days
Abdi et al. [43] (untreated sorghum)	7	8	12	3	21	42%-28 days
Abdi et al. [43] (untreated wheat and parboiled sorghum)	3	12	12	3	21	75%-28 days
Abdi et al. [43] (water, cracked maize, and parboiled barley)	0	15	12	3	21	100%-28 days
Ahmadivand et al. [87] (10 ng)	6	84	51	39	30	88%-30 days
Ahmadivand et al. [87] (2 ng)	12	78	51	39	30	76%-30 days
Ahmadivand et al. [87] (5 ng)	6	84	51	39	30	88%-30 days
Bunning et al. [89] (i.m. DNA vaccine with adjuvant)	4	6	10	0	70	60%-14 days
Bunning et al. [89] (i.m. DNA vaccine)	5	4	10	0	70	44%-14 days
Bunning et al. [89] (oral microencapsulated DNA vaccine)	10	0	10	0	70	0%-14 days
Cai et al. [90]	7	93	94	6	49	92%-14 days
Chen et al. [91] (pcDNA-RpsA)	8	22	29	1	35	71%-14 days
Chen et al. [91] (pcDNA-RplL)	6	24	29	1	35	78%-14 days
Citarasu et al. [92]	18	72	90	0	40	80%-10 days
Echeonwu et al. [52]	0	50	20	0	14	100%-10 days
Gong et al. [100]	8	17	25	0	14	68%-15 days
Habibi et al. [54] (feed)	7	8	15	0	14	53%-10 days
Habibi et al. [54] (water)	0	15	15	0	14	100%-10 days
Habibi et al. [55] (coated on the oiled wheat)	0	15	15	0	14	100%-17 days
Habibi et al. [55] (water)	0	15	15	0	14	100%-17 days
Huang et al. [101]	0	10	3	7	16	100%-10 days
Jeong et al. [58]	0	20	20	0	14	100%-7 days
Liu et al. [103]	18	22	40	0	42	55%-15 days
Omony et al. [71] NDV-173/UG/MU/2011	10	0	10	0	21	0%-14 days
Omony et al. [71] NDV-133/UG/MU/2011	4	6	10	0	21	60%-14 days
Omony et al. [71] 2014 NDV-177/UG/MU/2011	5	5	10	0	21	50%-14 days
Omony et al. [71] NDV-178/UG/MU/2011	8	2	10	0	21	20%-14 days
Pasnik and Smith [105] (25 ng-i.p)	88	22	110	0	90	20%-36 days
Pasnik and Smith [105] (25 ng-i.m)	22	88	110	0	90	80%-36 days
Pasnik and Smith [105] (5 ng-i.m)	110	0	110	0	90	0%-36 days
Pasnik and Smith [105] (50 ng-i.m)	11	99	110	0	90	90%-36 days
Ruan et al. 2020 [73]	0	12	12	0	21	100%-14 days
Sun et al. 2011 [107]	13	40	45	8	60	71%-20 days
Tan et al. [78] (IBV)	1	9	10	0	21	90%-14 days
Tan et al. [78] (NDV)	0	10	10	0	21	100%-14 days
Tu et al. [79] (0 days at 30–35 °C)	2	12	15	0	12	86%-14 days
Tu et al. [79] (10 days at 30–35 °C)	0	14	15	0	12	100%-14 days
Tu et al. [79] (17 days at 30–35 °C)	0	15	15	0	12	100%-14 days
Tu et al. [79] (24 days at 30–35 °C)	2	13	15	0	12	87%-14 days
Tu et al. [79] (3 days at 30–35 °C)	0	15	15	0	12	100%-14 days
Tu et al. [79] (eye drop)	1	9	10	0	14	90%-14 days
Tu et al. [79] (water)	1	9	10	0	14	90%-14 days
Wambura et al. [81] (feed)	0	15	15	0	35	100%-7 days
Wambura et al. [81] (wing web stab)	0	15	15	0	35	100%-7 days
Wang et al. [110]	9	31	40	0	35	77%-14 days
Xing et al. [112]	13	17	26	4	42	50%-15 days
Xu et al. [113]	1	29	13	17	60	92%-21 days
Xu et al. [114]	14	16	28	2	42	50%-15 days
Yi et al. [116]	11	19	30	0	30	63%-20 days
Yu et al. [117] (45 ng)	8	22	16	14	15	50%-21 days
Yu et al. [117] (90 ng)	4	26	16	14	15	75%-21 days
Zhang et al. [119] (pcDNA-M)	66	34	100	0	28	34%-20 days
Zhang et al. [119] (SWCNTs-pcDNA-M)	49	51	100	0	28	51%-20 days
Zhu et al. [120]	0	10	1	9	14	100%-10 days
Zhu et al. [121] (gavage)	13	17	30	0	21	57%-30 days
Zhu et al. [121] (mixed fodder)	11	19	30	0	21	63%-30 days

## Appendix A

**Table A1:** OIE listed diseases for which vaccines have been developed [13,14].

Name	Causative Agent	Disease Group
Anthrax	*Bacillus anthracis*	Multiple species diseases
Aujeszky’s disease	Suid herpesvirus 1 (SuHV-1)	Multiple species diseases
Brucellosis	*Brucella abortus*/*B. melitensis*	Multiple species diseases
Bluetongue	Bluetongue virus (BTV)	Multiple species diseases
Echinococcosis/hydatidosis	*Echinococcus granulosus*/*E. multilocularis*	Multiple species diseases
Epizootic haemorrhagic disease	Epizootic haemorrhagic disease virus (EHDV)	Multiple species diseases
Foot and mouth disease	Foot and mouth disease virus (FMDV)	Multiple species diseases
Heartwater (cowdriosis)	*Ehrlichia ruminantium* (formerly *Cowdria ruminantium)*	Multiple species diseases
Tuberculosis	*Mycobacterium tuberculosis complex*	Multiple species diseases
Japanese encephalitis	Japanese encephalitis virus (JEV)	Multiple species diseases
Paratuberculosis	*Mycobacterium avium* subsp. *paratuberculosis* (MAP)	Multiple species diseases
Q fever (or Coxiellosis)	Coxiella burnetii	Multiple species diseases
Rabies	Rabies virus (RABV) and other lyssaviruses	Multiple species diseases
Rift Valley fever	Rift Valley fever virus (RVF)	Multiple species diseases
Rinderpest	Rinderpest virus (RPV)	Multiple species diseases
Tularemia	*Francisella tularensis*	Multiple species diseases
West Nile Fever	West Nile virus (WNV)	Multiple species diseases
Bovine anaplasmosis	*Anaplasma marginale*/*A. centrale*	Bovinae
Bovine babesiosis	*Babesia bovis*/*B. bigemina*/*B. divergens*	Bovinae
Bovine genital campylobacteriosis (bovine venereal campylobacteriosis)	*Campylobacter fetus* subsp. *Venerealis*	Bovinae
Bovine viral diarrhoea	Bovine viral diarrhoea virus (BVDV)	Bovinae
Contagious bovine pleuropneumonia	*Mycoplasma mycoides* subsp. *Mycoides*	Bovinae
Haemorrhagic septicaemia	*Pasteurella multocida*	Bovinae
Infectious bovine rhinotracheitis/infectious pustular vulvovaginitis (IPV)	Bovine herpesvirus 1 (BoHV-1)	Bovinae
Lumpy skin disease virus	Lumpy skin disease virus (LSDV)	Bovinae
Theileriosis	*Theileria annulata* and *T. parva*	Bovinae
Trichomonosis	*Tritrichomonas foetus*	Bovinae
Enzootic abortion of ewes (ovine chlamydiosis)	*Chlamydia abortus*	Caprinae
Contagious agalactia	*Mycoplasma agalactiae* (Ma)	Caprinae
Contagious caprine pleuropneumonia	*Mycoplasma capricolum* subsp. *capripneumoniae* (Mccp)	Caprinae
Nairobi sheep disease	*Nairobi sheep disease virus* (NSDV)	Caprinae
Peste des petits ruminants virus	Small Ruminant Morbillivirus (SRMV)	Caprinae
Salmonellosis	*Salmonella abortusovis*	Caprinae
Sheep pox and goat pox	Sheeppox virus (SPPV) and goatpox virus (GTPV)	Caprinae
African horse sickness (AHS)	African horse sickness virus (AHSV)	Equidae
Equine rhinopneumonitis	Equid herpesvirus-1	Equidae
Equine viral arteritis (EVA)	Equine arteritis virus (EAV)	Equidae
Equine encephalomyelitis (Eastern, Western, Venezuelan) (EEE, WEE and VEE)	Equine encephalomyelitis viruses (Eastern, Western, Venezuelan) (EEEV, WEEV and VEEV)	Equidae
Equine influenza	Equine influenza viruses (H7N7, formerly equi-1, and H3N8, formerly equi2)	Equidae
Classical swine fever virus	Classical swine fever virus (CSFV)	Suidae
Nipah virus encephalitis	Nipah virus (NiV)	Suidae
Porcine reproductive and respiratory syndrome (PRRS)	Porcine reproductive and respiratory syndrome virus (PRRSV)	Suidae
Porcine cysticercosis	*Taenia solium*	Suidae
Transmissible gastroenteritis (TGE)	Transmissible gastroenteritis virus (TGEV)	Suidae
Camelpox	Camelpox virus	Other diseases
Leishmaniosis	*Leishmania* species (approximately 20 recognised)	Other diseases
Infectious salmon anaemia virus (Inf. with) (HPR-deleted or HPR0 genotypes)	Infectious salmon anaemia virus (ISAV)	Diseases of fish
Koi herpesvirus (Inf. with)	Koi herpesvirus (KHV)	Diseases of fish
Red sea bream iridovirus (Inf. with)	Red sea bream iridovirus RSIVD	Diseases of fish
Salmonid alphavirus (Inf. with)	Salmonid alphavirus (SAV)	Diseases of fish
Avian infectious bronchitis	Gammacoronavirus infectious bronchitis virus (IBV)	Aves
Avian infectious laryngotracheitis	Gallid alphaherpesvirus 1	Aves
Avian influenza	Low and High pathogenicity avian influenza viruses	Aves
Avian mycoplasmosis (M.synoviae)	*Mycoplasma synoviae*	Aves
Avian mycoplasmosis (Mycoplasma gallisepticum)	*Mycoplasma gallisepticum*	Aves
Duck virus hepatitis	Duck hepatitis A virus (DHAV)	Aves
Fowl typhoid	*Salmonella Gallinarum*	Aves
Infectious bursal disease (Gumboro disease)	Infectious bursal disease virus (IBDV)	Aves
Newcastle disease	Newcastle disease virus (NDV)	Aves
Pullorum disease	*Salmonella Pullorum*	Aves
Turkey rhinotracheitis	Avian metapneumovirus (Ampv)	Aves
Myxomatosis	Myxoma virus (MYXV)	Leporidae
Rabbit haemorrhagic disease	Rabbit haemorrhagic disease virus (RHDV)	Leporidae

**Table A2:** Information on animal vaccines production worldwide as submitted to the OIE by Member Countries. It is important to consider that not all the available vaccines on the market are reported as only the laboratories under national veterinary services are requested to provide information on the vaccines produced (e.g., vaccines produced by private industries might not be reported to the OIE).

Disease	Conjugate Vaccine	DNA Vaccine	Inactivated Vaccine	Live Attenuated Vaccine	Recombinant Vector Vaccine	Subunit Vaccine
African horse sickness				×		
Anthrax			×	×		
Aujeszky’s disease	×		×	×	×	
Avian infectious bronchitis	×		×	×	×	
Avian infectious laryngotracheitis	×		×	×	×	
Avian mycoplasmosis (*M. gallisepticum*)			×	×		
Bluetongue			×	×		
Bovine anaplasmosis			×	×		
Bovine babesiosis			×	×		
Bovine brucellosis			×	×		
Bovine viral diarrhoea			×	×		
Brucellosis (Brucella abortus)				×		
Brucellosis (Brucella melitensis)			×	×		
Camelpox			×	×		
Caprine and ovine brucellosis (excluding *B. ovis*)			×	×		
Classical swine fever			×	×	×	×
Contagious agalactia			×	×		
Contagious bovine pleuropneumonia				×		
Contagious caprine pleuropneumonia			×	×		
Duck virus enteritis			×	×		
Duck virus hepatitis			×	×		
Enterovirus encephalomyelitis			×	×		
Enzootic abortion of ewes (ovine chlamydiosis)			×			
Equid herpesvirus-X (EHV-X) (Infection with)			×			
Equine encephalomyelitis (Eastern)			×	×		
Equine encephalomyelitis (Western)			×			
Equina influenza			×	×		
Equine rhinopneumonitis			×			
Equine viral arteritis			×			
Foot and mouth disease			×	×		×
Fowl cholera			×	×		
Fowl typhoid			×	×		
Haemorrhagic septicaemia			×	×		
Highly pathogenic avian influenza			×	×	×	
Highly pathogenic influenza A viruses (infection with) (non-poultry incluiding wild birds)			×			
Infection with salmonid alphavirus		×				
Infectious bovine rhinotracheitis/infectious pustular vulvovaginitis	×		×	×	×	
Infectious bursal disease (Gumboro disease)	×		×	×	×	
Infectious haematopoietic necrosis		×				
Infectious pancreatic necrosis			×			×
Infectious salmon anaemia			×			
Japanese encephalitis				×		
Low pathogenic avian influenza (poultry)			×			
Lumpy skin disease			×	×		
Marek’s disease			×	×		
Myxomatosis				×		
Newcastle disease			×	×	×	
Ovine epididymitis (Brucella ovis)				×		
Peste des petits ruminants			×	×		
Porcine reproductive and respiratory syndrome			×	×	×	×
Pullorum disease			×			
Rabbit haemorrhagic disease			×	×		
Rabies			×	×	×	
Red sea bream iridoviral disease			×			
Rift Valley fever			×	×		
Rinderpest			×	×		
Salmonellosis (*S. abortusovis*)			×	×		
Sheep pox and goat pox			×	×		
Theileriosis				×		
Transmissible gastroenteritis			×	×		
Trichomonosis			×			
Turkey rhinotracheitis			×	×		
Venezuelan equine encephalomyelitis			×	×		
Vesicular stomatitis			×			
West Nile Fever			×			

The most produced type is the inactivated vaccine (available for 56/67 diseases), followed by the live attenuated vaccine (available for 49/67 diseases), recombinant vector vaccine (available for 10/67 diseases), conjugate vaccine (available for 5/67 diseases), subunit vaccine (available for 4/67 diseases), and DNA vaccine (available for 2/67 diseases) (supplementary material: Table A2). Generally, live-attenuated vaccines are more heat sensitive to potency loss during storage and distribution, thus requiring particular attention to maintain the cold chain. Conversely, inactivated and subunit vaccines can be particularly freeze sensitive, while DNA vaccines are very stable and do not require a cold chain.
